# Domain Wall Motion and the Interfacial Dzyaloshinskii–Moriya Interaction in Pt/Co/RuO_2_(Ru) Multilayers

**DOI:** 10.3390/ma18174008

**Published:** 2025-08-27

**Authors:** Milad Jalali, Kai Wang, Haoxiang Xu, Yaowen Liu, Sylvain Eimer

**Affiliations:** 1 School of Physics Science and Engineering, Tongji University, Shanghai 200092, China; miladj@tongji.edu.cn (M.J.); 2332714@tongji.edu.cn (H.X.); 2 National Key Laboratory of Spintronics, Hangzhou International Innovation Institute, Beihang University, Hangzhou 311115, China; 18253222718@163.com

**Keywords:** Dzyaloshinskii–Moriya interaction, magnetic multilayer, domain wall dynamics, spintronics

## Abstract

The interfacial Dzyaloshinskii–Moriya interaction (DMI) plays a pivotal role in stabilising and controlling the motion of chiral spin textures, such as Néel-type bubble domains, in ultrathin magnetic films—an essential feature for next-generation spintronic devices. In this work, we investigate domain wall (DW) dynamics in magnetron-sputtered Ta(3 nm)/Pt(3 nm)/Co(1 nm)/RuO_2_(1 nm) [Ru(1 nm)]/Pt(3 nm) multilayers, benchmarking their behaviour against control stacks. Vibrating sample magnetometry (VSM) was employed to determine saturation magnetisation and perpendicular magnetic anisotropy (PMA), while polar magneto-optical Kerr effect (P-MOKE) measurements provided coercivity data. Kerr microscopy visualised the expansion of bubble-shaped domains under combined perpendicular and in-plane magnetic fields, enabling the extraction of effective DMI fields. Brillouin light scattering (BLS) spectroscopy quantified the asymmetric propagation of spin waves, and micromagnetic simulations corroborated the experimental findings. The Pt/Co/RuO_2_ system exhibits a Dzyaloshinskii–Moriya interaction (DMI) constant of ≈1.08 mJ/m^2^, slightly higher than the Pt/Co/Ru system (≈1.03 mJ/m^2^) and much higher than the Pt/Co control (≈0.23 mJ/m^2^). Correspondingly, domain walls in the RuO_2_-capped films show pronounced velocity asymmetry under in-plane fields, whereas the symmetric Pt/Co/Pt shows negligible asymmetry. Despite lower depinning fields in the Ru-capped sample, its domain walls move faster than those in the RuO_2_-capped sample, indicating reduced pinning. Our results demonstrate that integrating RuO_2_ significantly alters interfacial spin–orbit interactions.

## 1. Introduction

The Dzyaloshinskii–Moriya interaction (DMI), originally proposed to explain weak ferromagnetism in antiferromagnets [1,2], has become central to modern spintronics due to its role in stabilising chiral spin textures such as Néel-type domain walls (DWs) [3,4,5,6,7] and magnetic skyrmions [8,9,10,11]. In ultrathin ferromagnetic films, interfacial DMI, arising from strong spin–orbit coupling and broken inversion symmetry at heavy metal interfaces, promotes preferred DW chirality [4,5,12]. This chirality enhances the efficiency of spin–orbit torque-driven DW motion [5,13], which is crucial for proposed applications such as racetrack memory and spin-based logic devices [14,15,16]. Quantifying and controlling DMI strength are thus critical for optimising device performance. Various experimental techniques have been developed, including DW motion under applied in-plane fields [5,17,18], the direct imaging of DW chirality [11,19], and the analysis of spin wave nonreciprocity using Brillouin light scattering (BLS) [20,21,22]. While bubble expansion methods provide straightforward estimates of effective DMI fields, BLS offers a direct and quantitative measure by probing frequency asymmetries of counter-propagating spin waves [21,23].

Recent studies have highlighted that subtle modifications at the ferromagnet interface can significantly alter DMI strength [5,8,24]. The insertion of ultrathin spacer layers—such as Ir or AlO_x_—between a heavy metal and ferromagnet has been shown to modify both the magnitude and sign of the DMI [8,20]. For example, an Ir layer inserted between Pt and Co alters effective DMIs while preserving left-handed DW chirality [8]. Similarly, buffer layers during growth affect interface quality and thus impact the DMI [24]. Such sensitivity underscores the importance of systematic investigations into how different capping or insertion layers influence the interfacial DMI, which can guide material engineering for efficient chiral spintronic devices.

Alongside developments in chiral magnetism, the recently proposed concept of altermagnetism has attracted considerable interest [25]. Altermagnets exhibit zero-net magnetisation and yet show significant spin splitting in their band structures due to unconventional symmetry breaking [26]. Ruthenium dioxide (RuO_2_) has been proposed as a candidate altermagnet, with theoretical predictions of large spin-split bands and d-wave spin textures. Experimental evidence supporting altermagnetism in RuO_2_ includes anomalous Hall effects and spin-resolved photoemission signatures [27]. However, recent high-resolution studies have questioned this interpretation, finding no observable spin splitting in RuO_2_ [28]. Thus, the existence of altermagnetism in RuO_2_ remains under debate. Although our study does not directly investigate altermagnetism, the potential exotic properties of RuO_2_ motivate interest in understanding its interfacial influence on magnetic behaviour, particularly regarding the DMI and domain wall dynamics.

In this work, we systematically investigate the effect of the interfacial DMI on bubble-shaped domain wall motion in Ta/Pt/Co multilayers capped with either RuO_2_ or Ru. Magnetic properties were characterised by vibrating sample magnetometry (VSM), while field-driven DW dynamics were studied via polar Kerr microscopy under varying in-plane magnetic fields. Complementary BLS measurements quantitatively determined the DMI constants. Additionally, micromagnetic simulations were carried out to model the domain wall creep and depinning behaviour, validating experimental observations. Our study reveals that introducing a RuO_2_ capping layer significantly modifies DMI strength compared to metallic Ru, with important implications for designing chiral spintronic devices.

From a technological standpoint, integrating oxide layers such as RuO_2_ into heavy metal/ferromagnet stacks offers several key advantages for chiral spintronic devices. Firstly, oxide capping layers are compatible with standard CMOS fabrication processes, facilitating seamless integration into existing semiconductor technology platforms. Secondly, oxide interfaces enable the possibility of electric field control over the interfacial DMI, allowing the low-power, voltage-controlled manipulation of chiral spin textures—a highly desirable capability for energy-efficient spintronic devices. Thirdly, oxide-induced modifications of interfacial spin–orbit coupling provide enhanced stability and tunability: by careful interface engineering, one can fine-tune the magnitude and even the sign of the DMI, thereby tailoring domain wall dynamics and improving skyrmion’s stability. Finally, RuO_2_-based multilayers demonstrate competitive performance, achieving DMI strengths comparable to those in Pt/Co/Ir or Pt/Co/AlO_x_ systems. This combination of CMOS compatibility, electrical tunability, improved interfacial control, and robust DMI positions RuO_2_-capped stacks as a promising platform for next-generation spintronic applications that require stable and controllable chiral spin textures. These compelling technological prospects served as a key motivation for us to investigate domain wall dynamics in Pt/Co multilayers capped with Ru versus RuO_2_ [5,8,29,30,31]. Our work provides a direct RuO_2_-vs.-Ru comparison on Pt/Co, quantifies the DMI coefficient by both BLS and DW dynamics, and interprets the difference through oxide-enhanced interfacial SOC, supported by micromagnetic modelling.

## 2. Experiment

**Figure 1:**
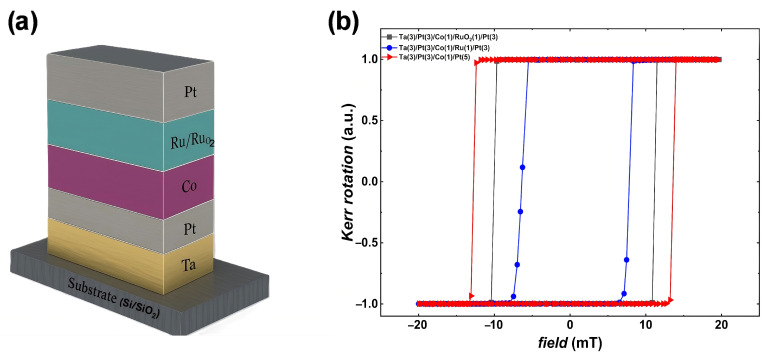
(**a**) Schematic of thin-film structure. (**b**) P-MOKE measurement for Pt(3)/Co(1)/Pt(5) and same multilayer with Ru and RuO_2_ as non-magnetic spacer.

Multilayer thin-film stacks Ta(3)/Pt(3)/Co(1)/RuO_2_(1)/Pt(3), Ta(3)/Pt(3)/Co(1)/Ru (1)/Pt(3) and Ta(3)/Pt(3)/Co(1)/Pt(5), (thickness in nanometers) were deposited on a 1 cm × 1 cm Si [001]/SiO_2_ substrate by a magnetron sputtering (Truth Equipment MS-700, Truth Equipment Co., LTD., Hefei, China). The vacuum before argon gas introduction was 10^−9^ mbar and the distance between the target and the substrates was around 20 cm. The flow of argon gas was fixed at 20 sccm. All layers were deposited using a DC power source. The layer thickness and roughness were controlled by XRR and AFM. All sputtering targets were of high purity (e.g., 99.99% for Pt and Ru) and depositions were carried out in high-purity Ar (99.99%) with a base pressure of 10^−9^ mbar; We also used a RuO_2_ target. A schematic of the thin-film structure is shown in Figure 1a and growth parameters are shown in Table 1. A 1 nm Co layer was used to ensure perpendicular anisotropy and a prominent interfacial DMI, as this thickness is known to support Néel DWs in Pt/Co-based multilayers. The spacer/capping layer thickness was fixed at 1 nm for both Ru and RuO_2_ to provide a single-atomic interface with Co while minimising total film thickness (for consistent demagnetising effects). Thicker RuO_2_ might further alter properties, but 1 nm is sufficient to form a continuous layer and is chosen to parallel typical heavy metal interlayer studies (e.g., 1 nm Ir in Pt/Co/Ir) [8]. Pt capping in the control was 5 nm (versus 3 nm in the others) to keep the total stack thickness similar and ensure that Co in all samples had a protected top surface. The experiments were performed at room temperature.

## 3. Results and Discussion

### 3.1. Magnetic Characterisation

Figure 1b shows the P-MOKE hysteresis loops for the multilayer with different spacer layers: Ta(3)/Pt(3)/Co(1)/Ru(1)/Pt(3), Ta(3)/Pt(3)/Co(1)/RuO_2_(1)/Pt(3), and Ta(3)/Pt(3)/Co(1)/Pt(5). The Kerr rotation versus out-of-plane magnetic field reveals square loops characteristic of strong perpendicular magnetic anisotropy (PMA). Extracted coercive fields (*Hc*) are 7.46 mT, 10.61 mT, and 13.18 mT, respectively. The remanent Kerr rotation nearly equals saturation, indicating abrupt switching. The RuO_2_-capped sample exhibits a broader switching region, likely due to modified interfacial exchange or increased structural disorder introduced by the oxide layer.

**Figure 2:**
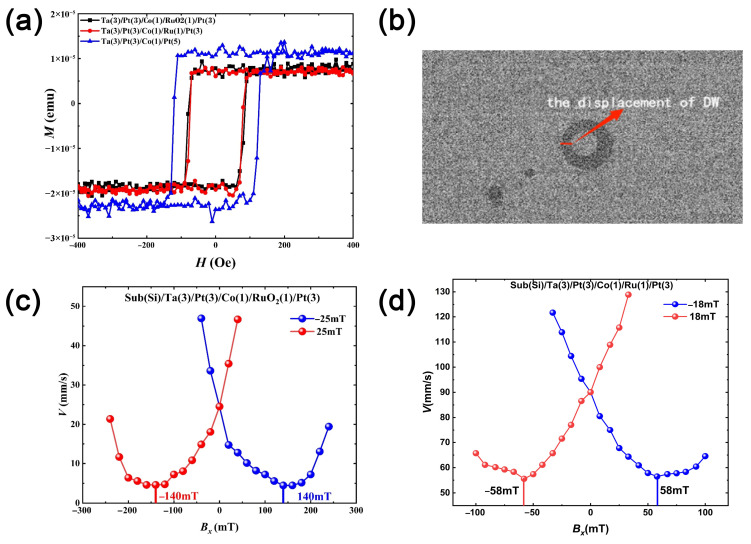
(**a**) The magnetic hysteresis loop of the thin film. (**b**) The measurement of DW velocity using domain wall displacement. (**c**,**d**) Domain wall velocity versus *B_x_* (the in-plane field) for (**c**) Ta(3)/Pt(3)/Co(1)/RuO_2_(1)/Pt(3) and (**d**) Ta(3)/Pt(3)/Co(1)/Ru(1)/Pt(3).

VSM measurements confirmed these behaviours, with saturation magnetisation Ms calculated by dividing the saturation moment by the Co layer volume (*M*_s_ = *M*/*V*). Figure 2a compares magnetic hysteresis loops of the three stacks, confirming PMA (out-of-plane magnetic anisotropy) and allowing an assessment of coercivity and saturation across configurations. Interface quality strongly influences magnetic properties. The broader switching and reduced domain wall velocities observed in RuO_2_-capped samples suggest increased pinning due to partial oxidation, intermixing, or roughness at the Co/RuO_2_ interface. Micromagnetic simulations (which will be discussed in detail in the Simulations Section) incorporating grain structure and local anisotropy fluctuations confirm that such heterogeneities enhance domain wall pinning and lead to non-uniform motion. Finite-size effects and lateral confinement further amplify these phenomena, emphasising the need for the precise control of deposition and oxidation conditions to ensure reproducible device performance.

### 3.2. Domain Wall Dynamics and DMI Extraction

Field-driven domain wall (DW) dynamics were studied by Kerr microscopy. After saturating magnetisation out of plane, reversed magnetic domains were nucleated by applying magnetic field pulses. DW velocity was determined by measuring wall displacement during pulse duration (Figure 2b).

To extract the effective DMI field (*H_DMI_*), DW velocity was measured as a function of the static in-plane magnetic field (*B_x_*) applied along DW propagation.

This approach uses the asymmetric expansion behaviour caused by the DMI. This allows both the magnitude and the sign of the DMI constant, as well as domain wall chirality, to be determined.

The velocity reaches a minimum when *B_x_* compensates the DMI effective field, as shown in Figure 2c,d for RuO_2_- and Ru-capped samples. The minima occur near ±140 mT and ±58 mT, respectively, allowing the determination of DMI magnitude, sign, and DW chirality. Notably, DW velocity in the Ru-capped sample is significantly higher than in RuO_2_, despite smaller driving fields, indicating smoother interfaces and reduced pinning. We note that the minimum velocity at *B_x_* ≈ −B_DMI_ is a DMI-related dynamic effect and not caused by the sample’s static coercivity. In our measurements, reversed domains were pre-nucleated and then driven by short out-of-plane field pulses, chosen to translate the wall in the creep regime without fully switching the film. The sample’s coercive field therefore does not set the position of the minimum; instead, the minimum arises because, at *B_x_* ≈ −B_DMI_, the wall becomes Bloch-like, which maximises the elastic energy and the pinning barrier, suppressing creep velocity. Moving away from this compensation restores the chiral Néel character and the velocity increases. Even at *B_x_* ≈ −B_DMI_, the domain wall did not collapse; motion persisted at a very low rate. Upon shifting *B_x_* away from the compensation value, the velocity recovered, confirming that the minimum is reversible and governed by the DMI rather than irreversible pinning or hysteresis.

### 3.3. Brillouin Light Scattering (BLS) Spectroscopy

**Figure 3:**
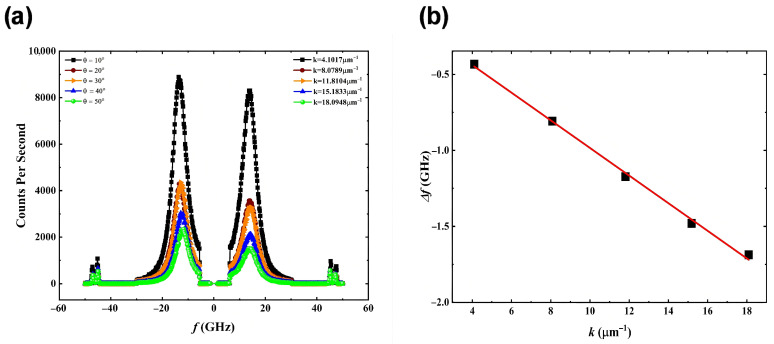
(**a**) The BLS spectra measured for Ta(3)/Pt(3)/Co(1)/RuO_2_(1)/Pt(3). (**b**) The frequency difference vs. wave vector. The red line is the fitting curve using formula ∆*f* = 2*γDk*/(π*M*_s_).

BLS measurements probed spin wave nonreciprocity to quantify the DMI. With an in-plane magnetic field of 1 T saturating magnetisation, frequency shifts Δ*f* between Stokes and anti-Stokes peaks were measured at various incidence angles (*θ* = 10° to 50°), corresponding to wave vectors *k* = 4.1–18.1 μm^−1^ (Figure 3a). The relation ∆*f* = 2*γDk*/(π*M*_s_), where *k* = (4π*/λ*) × sin *θ*, was used to extract DMI constant D via linear fitting (Figure 3b).

The measured DMI values are 1.082 mJ/m^2^ (Pt/Co/RuO_2_/Pt), 1.029 mJ/m^2^ (Pt/Co/Ru/Pt), and 0.232 mJ/m^2^ (Pt/Co/Pt). The stronger DMI observed in Co/RuO_2_ compared to Co/Ru can be attributed to enhanced interfacial SOC and inversion symmetry breaking. The presence of oxygen at the interface introduces a stronger interfacial electric field and allows for greater orbital hybridisation between the 3d states of Co and the 4d–2p hybridised states of RuO_2_, which has been shown to amplify DMI strength at similar metal/oxide interfaces [31,32].

The sign of the DMI favours left-handed Néel walls in RuO_2_- and Ru-capped samples, consistent with DW experiments. The measurable DMI in nominally symmetric Pt/Co/Pt samples highlights the extreme sensitivity of the DMI to atomic-scale interface asymmetries.

For comparison, Pt/Co/Ir multilayers typically exhibit DMI values of 1.0–1.5 mJ/m^2^ [8], and Pt/Co/AlO_x_ [5] systems can reach ~2 mJ/m^2^ depending on oxidation and interface quality. These benchmarks position RuO_2_-based stacks as competitive candidates for stabilising chiral spin textures, with the added advantage of tuneable interface chemistry.

## 4. Simulations

Using the open-source micromagnetic simulation software MuMax^3^, we explored how the interfacial Dzyaloshinskii–Moriya interaction (DMI) affects domain wall (DW) dynamics in ultrathin ferromagnets with perpendicular magnetic anisotropy (PMA). According to experimental observations reported by Je et al. [17], the dependence of DW velocity on the in-plane magnetic field arises primarily from variation in the depinning field, *H*_d_, as a function of the applied in-plane field, *H*_IP_. This occurs because the in-plane field modifies the internal structure and energy distribution of the domain wall, thereby altering its interaction with local pinning areas and ultimately shifting the critical field required for depinning. To reduce computational costs, we therefore modelled a 1 × 2 μm^2^ strip with 1 nm thickness. A mesh cell size of 2 × 2 × 1 nm^3^ was selected to ensure that the lateral mesh spacing remained below the exchange length of Co. Periodic boundary conditions were applied along the *y* axis to simulate an indefinitely broad film. Material properties such as anisotropy, exchange stiffness, and local thickness may vary at the nanoscale, thereby altering the micromagnetic energy landscape [27,33,34].

When a domain wall traverses such heterogeneous regions, it may be immobilised by local energy barriers, which are random in both magnitude and spatial distribution. This pinning mechanism has been experimentally confirmed in various systems, including Pt/Co/AlO_x_ multilayers [35]. To model the magnetic disorders, we used the Voronoi tessellation as a feature in MuMax^3^. The mean grain diameter was fixed at 10 nm, consistent with experimental observations [8]. Assuming an average grain diameter of 10 nm fully tiling the simulated strip of 1 × 2 µm^2^ (area ≈ 2.0 µm^2^), the corresponding grain count is on the order of N≈ 2.0 × 10^4^ (≈ 20,000 grains). Within each grain, anisotropy constant *K*_u_ was selected randomly from a uniform distribution centred at $K_{u}=K_{eff}+\frac{1}{2}\mu_{0}M_{s}^{2},$ with a variation of ±15%.

A domain wall was initialised at the centre of the strip (*x* = 0), with upward and downward magnetised domains on either side. This central placement is commonly adopted in micromagnetic simulations to avoid boundary-induced artefacts and isolate intrinsic DW dynamics. For each predetermined value of *H*_IP_, spatially uniform out-of-plane field *H*_OP_ (along the *z*-axis) was applied and incrementally raised by 2 mT. After each increment, the system was relaxed; the lowest *H*_OP_ that triggered rapid DW displacement was recorded as the depinning field, *H*_d_, for that *H*_IP_. This procedure was repeated 100 times to average out statistical fluctuations arising from disorder, and the average *H*_d_ was retained. At low *H*_OP_, DW velocity *v* follows the classical creep law [36]:

$$v(H_{op},T)=v\left(H_{d},T\right)exp\left(-\frac{\Delta E}{k_{B}T}\right),$$

where $v\left(H_{d},T\right)$ is the characteristic velocity at the depinning field, $k_{B}$ is Boltzmann’s constant, and *T* = 300 K, which was kept fixed during the simulation procedure. The pinning energy barrier is given by

$$\Delta E=k_{B}T_{d}\left({\left[\frac{H}{H_{d}}\right]}^{-\frac{1}{4}}-1\right),$$

with *T*_d_ representing the characteristic depinning temperature [37]. This universal relation holds across all studied magnetic systems. Once the applied field exceeds the intrinsic *H*_d_ of the film, pinning becomes negligible, and the DW’s velocity deviates from the creep law.

Experimentally, one of the simplest methods to quantify the DMI is to apply a series of in-plane fields, measuring the asymmetric creep velocities of DW expansion, and locate the minimum velocity [17,38]. In the absence of the DMI and *H*_IP_, DW magnetisation lies within the wall plane, forming a Bloch-type wall that minimises surface energy. When the DMI is significant, it manifests as an effective field, *H*_DMI_, oriented normal to the DW, forcing the magnetisation to align perpendicular to the wall plane. Its orientation is set by the chirality of the DMI [39], and the wall takes a Néel form.

**Figure 4:**
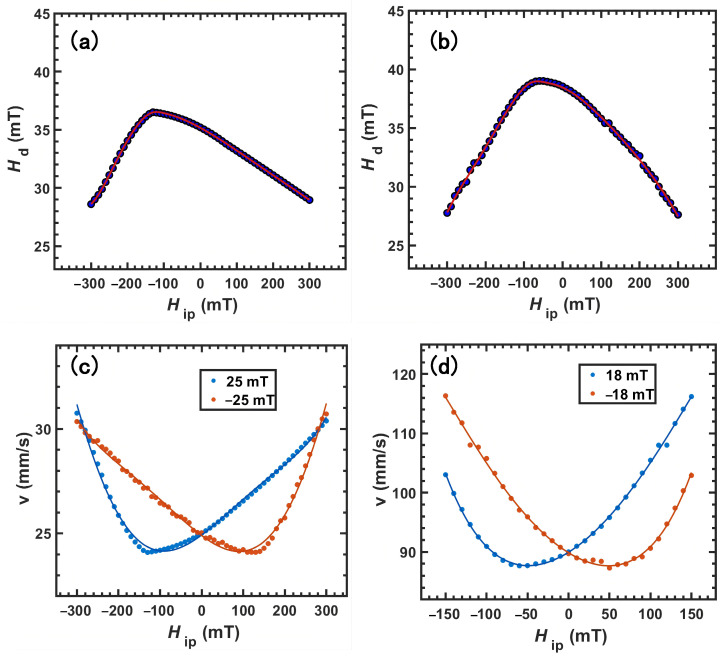
Depinning field as a function of in-plane applied field from micromagnetic simulations for RuO_2_ (**a**) and Ru (**b**). Domain wall velocity, *v*, as a function of in-plane applied field, *H*_IP_, for RuO_2_ (**c**) and Ru (**d**).

All simulation parameters were either directly derived from our present experiments or adopted from representative Pt/Co reference systems reported in the literature; the intention was to isolate and compare the intrinsic differences in domain wall dynamics arising from the two insertion-layer configurations [40]. For Sample I (with a RuO_2_ capping layer), the following values were used: *M*_s_ = 1.3 × 10^6^ A m^−1^, *K*_u_ = 1.6 × 10^6^ J m^−3^ and *D* = −1.1 × 10^−3^ J m^−2^. For Sample II (with a Ru insertion layer), the parameters were *M*_s_ = 1.5 × 10^6^ A m^−1^, *K*_u_ = 1.8 × 10^6^ J m^−3^, and *D* = −0.85 × 10^−3^ J m^−2^. Exchange stiffness *A*_ex_ = 2.2 × 10^−11^ J m^−1^ and Gilbert damping factor *α* = 0.03 were kept identical in all cases [41]. Figure 4a,b illustrate the resulting *H*_IP_–*H*_d_ graph for the two parameter sets. The graphs are clearly asymmetric about *H*_IP_ = 0, and the degree of asymmetry increases with the magnitude of *D*. The DW’s velocity and pinning potential are both affected by a bigger *D*. The velocity exhibits a minimum when H_IP_ compensates H_DMI_; at this compensation field, the wall is Bloch-like and its energy is maximised, which raises the pinning barrier and thus suppresses creep velocity.

Thus, although an in-plane field cannot drive a DW directly in a PMA film, it can still modulate the velocity by rotating the wall’s magnetisation. With both *H*_OP_ and *H*_IP_ applied, the magnitude and sign of *H*_DMI_ can be extracted by identifying the *H*_IP_ value that yields the velocity minimum at a fixed out-of-plane field. The relevant *H*_IP_-*v* curves from the simulated *H*_IP_–*H*_d_ relations were derived. By correlating the experimentally observed *v*(*H*_OP_) with the calculated *H*_d_ values, we derived accurate *v*_0_ and *T*_d_. The computed velocities nearly align with the experimental data, and the positions of the minima correspond well with theoretical predictions. As can be seen in Figure 4a,b, the graphs are clearly asymmetric about *H*_IP_ = 0, and the degree of asymmetry increases with the magnitude of *D*. This asymmetry is intrinsic to interfacial DMI systems. The DMI selects a chiral Néel wall with internal magnetisation; *H*_IP_ parallel (antiparallel) to this internal magnetisation lowers (raises) the wall’s energy and the elastic stiffness, thereby reducing (increasing) the depinning field, *H*_d_, and the creep barrier. The velocity minimum occurs near *H*_IP_ ≈ −*H*_DMI_, where the wall becomes Bloch-like and its energy is maximised.

## 5. Conclusions

In summary, we find that capping Pt/Co with RuO_2_ (1 nm) enhances the interfacial DMI by ~5% compared to a Ru (1 nm) cap (as evidenced by D_BLS_ ≈ 1.08 vs. 1.03 mJ/m^2^) and by nearly 5 times compared to symmetric Pt capping (D_BLS_ ≈ 0.23 mJ/m^2^). This stronger DMI correlates with pronounced asymmetry in domain wall motion under in-plane fields for the RuO_2_-capped stack. Despite slightly higher coercivity and more pinning in the oxide-capped films, the domain walls remain the chiral Néel-type and their dynamics are well-described by creep theory and micromagnetic simulations. These results demonstrate that integrating an oxide like RuO_2_ can tune the chiral spin interactions at the interface without an external field, offering a route to engineering the DMI in device structures. Importantly, RuO_2_-capped multilayers combine a strong DMI with practical advantages (CMOS compatibility and potential electric field control), underlining their promise for future spintronic applications. This work provides a foundation for using oxide layers to modulate the DMI and suggests follow-up investigations (e.g., with varying oxide thicknesses or interface treatments) to further optimise domain wall behaviour.

## Figures and Tables

**Table 1:** The growth parameters of the sample.

Layer	Vacuum (mbar)	Power (W, DC)	Growth Rate (s/nm)
Ta	5.3 × 10^−3^ mbar	50	47
Pt		25	32
Co		25	75
RuO_2_		25	92
Ru		50	29

## Data Availability

The original contributions presented in this study are included in the article. Further inquiries can be directed to the corresponding authors.
